# Effect of psychosocial interventions on the quality of life of patients with colorectal cancer: a systematic review and meta-analysis

**DOI:** 10.1186/s12955-018-0943-6

**Published:** 2018-06-08

**Authors:** Heesook Son, Youn-Jung Son, Hyerang Kim, Yoonju Lee

**Affiliations:** 1 Red Cross College of Nursing, Chung-Ang University, 84 Heukseok-Ro, Dongjak-Gu, Seoul, 06974 Republic of Korea; 2 College of Nursing, Pusan National University, 49 Busandaehak-ro, Mulgeum-eup, Yangsan-si, Gyeongsangnam-do 50612 Republic of Korea

**Keywords:** Colorectal cancer, Psychosocial interventions, Quality of life, Meta-analysis

## Abstract

**Background:**

We conducted a systematic review and meta-analysis of randomized controlled trials examining the effect of psychosocial interventions on the quality of life of patients with colorectal cancer.

**Methods:**

We searched the main health-related databases for relevant papers. Then, we examined the titles and abstracts of the retrieved papers, applying exclusion criteria to filter out irrelevant papers; a more in-depth filtering process was then conducted by reading the full texts. Eight studies remained at the end of this process. Next, we performed data extraction and assessed the methodological quality of the selected studies. This was followed by computation of effect sizes and the heterogeneity of the results, and then an assessment of the potential bias.

**Results:**

The systematic review found that most of the interventions in these eight studies did not have a significant effect on quality of life. Meanwhile, the meta-analysis, the overall effect of psychosocial interventions at the post-intervention period was found to be statistically significant but small.

**Conclusions:**

This meta-analysis provides evidence for the beneficial effect of face-to-face psychosocial interventions on the quality of life of colorectal cancer patients. It is, however, suggested that further studies be conducted on this topic to assess the roles of physical functioning and severity of symptoms before utilizing such face-to-face interventions.

**Electronic supplementary material:**

The online version of this article (10.1186/s12955-018-0943-6) contains supplementary material, which is available to authorized users.

## Background

Colorectal cancer (CRC) is a global concern and one of greatest causes of death in developed and developing countries. It is the third most common cancer afflicting both men and women, with 1.4 million cases and 693,900 deaths estimated to occur annually [1]. In recent decades, although the incidence rate has increased, CRC mortality rates have decreased in many countries [1, 2]. It is widely believed that regular screening and improved treatments are the main contributors to this decrease in mortality [3, 4].

Throughout the disease trajectory, cancer patients experience psychological distress, including depression and anxiety [5], which in turn lead to worsened quality of life [6]. Furthermore, undergoing surgical treatments such as stoma formation can decrease psychosocial health and quality of life even further, particularly in patients with CRC [7–10]. Both psychological distress and a lack of social support have significant negative impacts on the quality of life of patients with CRC. For example, one previous study found that psychological distress was a significant predictor of both the mental and physical health domains of quality of life [11]. Conversely, another study found that patients with greater social support had better quality of life at one year post-surgery [12]. These results suggest that implementing psychosocial interventions might be crucial for improving the quality of life of patients with CRC.

The term “psychosocial intervention” encompasses interventions that include psychological and social contents; specifically, “psychosocial” is defined by the Oxford English Dictionary as “involving the influence of social factors of human interactive behavior.” Psychoeducational programs are frequently provided for cancer patients in an attempt to improve their quality of life and to provide them with further information on the various methods of coping with cancer that they can perform in daily life. These programs have shown a positive effect on health-related quality of life [13–15]. Over the last decade, various types of psychosocial interventions have been conducted with the aim of improving CRC patients’ quality of life. Literature reviews seeking to synthesize the evidence concerning the effects of such psychosocial interventions on the outcomes of patients with CRC were also rigorously conducted during this same period. These comprehensive reviews determined that various types of interventions, including home visits, telephone sessions, and individual/group sessions, clearly reduce psychological distress and increase quality of life [16].

Most recently, in 2017, a systematic review of 14 randomized controlled trials of psychosocial interventions for CRC patients was published [17]; however, this study did not include a meta-analysis, which would enable us to quantify the strength of the evidence from previous studies. This would have clear clinical implications for healthcare providers by enabling them to recognize which interventions are most effective. Since a meta-analysis provides more statistically robust and reliable findings [18], it can help healthcare providers choose more effective and practical interventions for use in the field.

Meta-analyses have been included in past studies investigating the effects of psychosocial interventions on cancer survivors’ quality of life, revealing that such psychosocial interventions have both short- (less than eight months) and long-term (greater than eight months) effects on quality of life [19] and that psychosocial intervention with durations of over 12 weeks are more effective than those with shorter durations [20]. However, to our knowledge, the effect of psychosocial interventions on the quality of life of CRC patients has not yet been thoroughly analyzed. The abovementioned meta-analyses aggregated all types of cancer patients in their analyses, and therefore the results may differ from those for patients with CRC.

Little is known about the effectiveness of a variety of psychosocial interventions. The aims of this study are to present the results of a systematic review and meta-analysis of randomized controlled trials examining the effects of psychosocial interventions on the quality of life of CRC patients.

## Methods

### Literature search

The present study was conducted in accordance with the PRISMA guidelines for systematic reviews and meta-analyses. The articles were identified using the main databases related to health, including PubMed, EMBASE, CINAHL, the Cochrane database, PsycINFO, Web of Science, and SCOPUS, with the search criteria set to identify English-language, peer-reviewed studies published between January 2000 and October 2016. We performed searches using the combinations of the following keywords and similar terms (consisting of MeSH and entry terms): “colorectal cancer,” “colorectal neoplasm,” “colorectal tumor,” “quality of life,” “wellbeing,” “psychological outcome,” “psychosocial therapy,” “psychosocial intervention,” “education,” “counseling,” and “behavioral therapy.” To avoid missing potentially applicable articles, comprehensive searches using these keywords and similar terms were conducted. The strategy was modified as appropriate for the different databases (see Additional file 1).

### Inclusion and exclusion criteria

The primary inclusion criteria were being a randomized controlled trial of a psychosocial intervention and focusing on quality of life as the outcome. We defined psychosocial interventions as interventions involving psychological or social support. For the psychological support interventions, we selected cognitive behavioral therapy, psychotherapy, counseling, supportive therapy, and motivational interviewing as relevant interventions. For the social support intervention, we included social-skills training, which focuses on developing social networks and training to minimize social isolation or conflict (familial/work). Another eligibility criterion was that the interventions had to have been delivered by trained personnel such as nurses, allied healthcare workers, or psychologists. A wide range of intervention delivery methods were included in the analysis: group vs. individual-focused and telephone/web-based vs. face-to-face or hybrid.

The exclusion criteria were as follows: 1) was not a randomized controlled trial; 2) did not measure quality of life as the outcome (e.g., studies that measured only psychological factors such as anxiety, depression, or stress were excluded); 3) did not use formal psychometric scales to assess quality of life; 4) unavailable full text; and 5) limited information for computing the common effect size.

### Data extraction and quality assessment

**Fig. 1:**
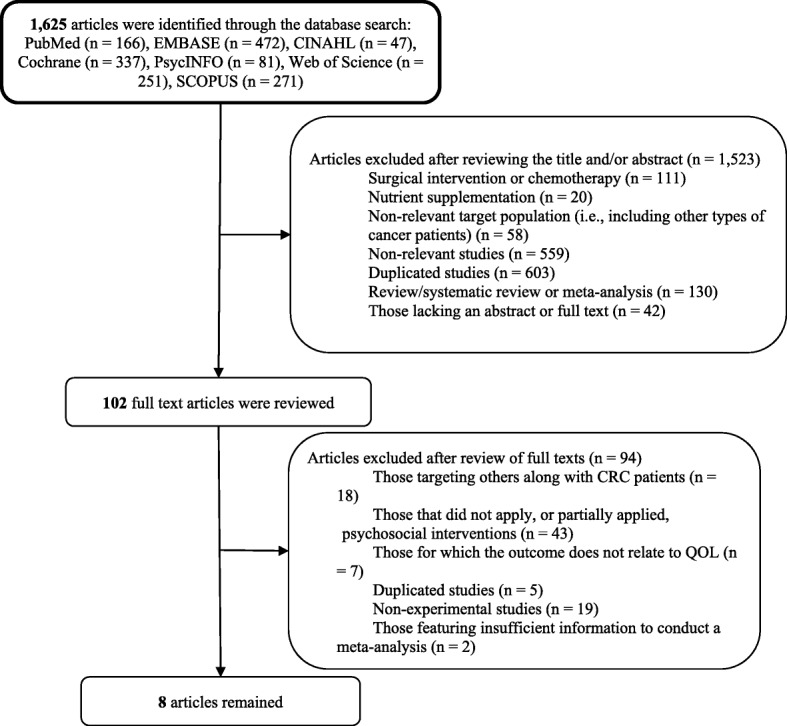
Plasma flow for literature search strategy

A total of 1625 articles were identified through the database search. Three authors then independently reviewed each title and abstract and compared their decisions; during this process, articles that used a less relevant study design (i.e., review papers) and those lacking abstracts or full texts (i.e., poster presentations) were excluded. Consequently, 102 full-text articles remained, and these advanced to the next stage of the filtering process, in which articles with meeting any of the following criteria were excluded: did not target patients with CRC only; did not apply, or only partially applied, psychosocial interventions; did not mention effects on quality of life in their outcomes; were duplicate studies; were non-experimental; and lacked sufficient information to facilitate a meta-analysis. Just eight studies advanced to the meta-analysis (see Fig. 1).

In the next stage, data regarding study design, participants, interventions, comparators, and outcomes were extracted using a predesigned data extraction form. Specifically, for study design, relevant data included the country in which the study took place, the sample size, and the timing of the follow-ups; data concerning participant characteristics included CRC diagnosis, gender, and age; data concerning intervention characteristics included information regarding components applied, personnel involved, and duration; and data concerning the outcomes included the means and standard deviations or number of events. In cases where published data was incomplete or unclear, the authors of the studies in question were contacted for clarification. All the above data were extracted independently by three reviewers, and any disagreements between two reviewers were resolved through discussion or by consulting the third reviewer.

Next, three reviewers (HS, YS, and HK) independently assessed methodological quality using the Cochrane Collaboration’s risk of bias assessment tool [21]. This tool assesses risk as being high, low, or unclear for the following domains: random-sequence generation, allocation concealment, blinding of outcome assessment, incomplete outcome data, and selective reporting and blinding of participants and personnel (administering the intervention). Due to the nature of the studies analyzed, the latter domain was not assessed in this review. Disagreements were resolved through discussion or consultation with another reviewer (YL).

### Statistical analysis

As there were variances in the values of the included studies, effect sizes were computed using Comprehensive Meta-Analysis software package Version 3.0 (trial). The effects of the psychosocial interventions were determined by applying Hedges’ g, which could be interpreted as small (g < 0.3), medium (g > 0.5), or large (g > 0.8) [22]. The 95% confidence intervals (CIs) were calculated using mean scores and standard deviations, change scores, or *p*- or t-values [23]. The heterogeneity of the results across studies was assessed using Q and I^2^ statistics, and any obvious heterogeneity was predefined as *p* < 0.05 in Q statistics or an I^2^ value of 50% or higher [24]; in cases where heterogeneity was found, a random-effects model was used.

Most of the studies had multiple outcomes; therefore, we calculated the overall effect size using the mean of the selected outcomes with a random-effects model, and then used a fixed-effects model to conduct subgroup analyses according to intervention type, length of the follow-ups implemented, and measurement tool. Next, potential publication bias was investigated by initially conducting a visual inspection of the funnel plots. Then, funnel plot asymmetry was tested using Egger’s test, and the trim and fill method was used to adjust for publication bias [25]. We also conducted sensitivity analysis to assess the influence of each study on the overall effect by removing one of the studies in each round.

## Results

### Characteristics and quality of included studies

Table 1 summarizes the descriptions of each selected study and the characteristics of the samples. Of the eight randomized controlled trials selected, which featured a cumulative total of 2117 patients, three and two studies were conducted in Australia and the United States, respectively, while the remaining three studies were performed in Hong Kong, Canada, and Denmark. The publishing years ranged from 2005 to 2016, with seven published in 2010 or later. Participants’ mean age ranged from 56.2 to 68.6 years, and women accounted for between 31.6 and 63% of the sample populations.

**Table 1:** Description of studies and sample characteristics (*n* = 2117)

	Subjects	Intervention	Outcome (tool)	Main results
(Carmack et al., 2011) in the US[27]	∙Individuals with stage I, II, III colon or rectal cancer (*n* = 40: intervention *n* = 25 vs. control *n* = 15) ∙Mean age = 56.2 years ∙Women (63%)	12 one-hour sessions over four months (nine weekly sessions, two bimonthly sessions, and one concluding session in month 4)	QOL (European Organization for Research and Treatment of Cancer (EORTC) questionnaires)	For individuals in the intervention group, EORTC emotional functioning was not found to have significantly improved at two months, but had at four months. For the control group, EORTC was not significant at two or four months.
(Jefford et al., 2016) in Australia[48]	∙Individuals with stage I, II, or III colon or rectal cancer (*n* = 217: survivor care *n* = 110 vs. normal care *n* = 107) ∙ Mean age = 62.1 years for the intervention group and 63.1 years for the control group ∙ Women (47.7% for the intervention group and 49.1 for the control group)	Four main components: 1) an information package; 2) a nurse-led, face-to-face end-of-treatment session; 3) a tailored survivor care plan; and 4) nurse-led telephone follow-ups at one, three, and seven weeks after the first intervention session	European Organization for Research and Treatment of Cancer core questionnaire (EORTC QLQ-30) and CRC module (EORTC QLQ CR-29)	At two and six months post-baseline, all differences in QLO-C30 and QLQ C-29 between the two groups were small and not significant.
(Lee, Ho, & Chan, 2010) in Hong Kong[49]	∙Patients with colorectal cancer (*n* = 166: intervention *n* = 84 vs. *n* = 82 control) ∙Mean age = 60 years (58.9 years for the intervention groups and 60.5 years for the control group) ∙Women (33.7 and 36.4% for the intervention groups and 31% for the control group)	Participants in intervention received body-mind-spirit intervention for 15 h. Each group consisted of 10 to 12 members and met weekly for five weeks.	QOL was measured using the validated Chinese version of the SF-36	No significant interaction effect (time × intervention) either in the intervention or control groups for SF-36 was found.
(Lepore, Revenson, Roberts, Pranikoff, & Davey, 2015) in the US[50]	∙Patients diagnosed with stage I-III cancer of the colon or rectum and who had completed surgical treatment within the previous five years (*n* = 193: intervention *n* = 101 vs. control *n* = 92) ∙ Mean age = 54.4 years for the intervention group and 55.8 years for the control group ∙ Women (49.5% for the intervention group and 48.9 for the control group)	Participants were asked to write for 15 min twice a week for two weeks about their deepest thoughts and feelings concerning their cancer, other stressors, or both.	Five domains of the Cancer Quality of Life questionnaire (QLQ-30) developed by the European Organization for Research and Treatment of Cancer	There was no statistical difference between the intervention and control groups in terms of: global quality of life; physical functioning; role functioning; emotional functioning; cognitive functioning; or social functioning.
(Ohlsson-Nevo et al., 2016) in Sweden[13]	∙Patients treated surgically for colon, rectal, or anal cancer (*n* = 86: intervention *n* = 47 vs. control *n* = 39) ∙ Mean age = 66.1 years for the intervention group and 65.9 years for the control group ∙ Women (41.7% for the intervention group and 31.6% for the control group)	The program included seven meetings that each featured 60-min informational lectures. Topics included colorectal cancer, music and relaxation, the operating theatre, the importance of engaging in physical activities, the meaning of food, crisis and crisis intervention, and patients’ organization	SF-36 were measured at baseline, one, six, and 12 months after the end of the program	∙The intervention group reported significantly better overall mental health status than the control at one month after the end of the program. The between-group difference was of moderate size. ∙No statistically significant between-group differences in overall physical health status were observed at any of the follow-up assessments, but the effect sizes indicated that the intervention group had a small effect after six and 12 months. ∙Within the intervention group, significant improvements in both PCS and MCS were found: PCS; 0-one month, zero–six months, zero–12 months; MCS zero–, zero–six months, zero–12 months.
(Ross, Thomsen, Karlsen, Boesen, & Johansen, 2005) in Denmark[51]	∙ Colorectal cancer patients (*n* = 249; intervention *n* = 125 vs. control *n* = 124) ∙Median age = 68.8 years for the intervention group and 68.1 years for the control group ∙Women (50% for the intervention group and 53% for the control groups)	Over the first two years after discharge, the intervention group received 10 home visits from a project nurse or a medical doctor. These visits were aimed at providing emotional and information and support and encouraging the patients to make use of their own social networks to cope with the disease.	European Organization for Research and Treatment of Cancer (EORTC) quality of life core questionnaire QOQ-C30, and EORTC QLQ-CR38 for the specific colorectal cancer module	∙At the three-month follow-up, following the most intensive period of intervention, the only significant difference between the intervention and control groups was symptoms of fatigue. ∙Also at the three-month follow-up, all 15 subscales/items of EORTC QLQ-C30 were in favor of the intervention group, but this pattern did not persist during the full two-year period of follow-ups and was not observed on the colorectal cancer module (EORTC QLQ-CR38). ∙The intervention had no overall effect on the level of QOL. Further, no differential development with time during follow-up was observed on the employed scales, except for a differential development with time in the groups for social functioning: at the first follow-up interview the control group had a lower level of social functioning than the intervention group, but this difference diminished with time.
(Young et al., 2013) in Australia [52]	∙Adult patients undergoing surgery for primary colorectal cancer (*n* = 756; intervention *n* = 387 vs. control *n* = 369) ∙Mean age = 68.6 years for the intervention group and 67 years for the control group ∙Women (43.2% for the intervention group and 45.8% for the control group)	This intervention involved no face-to-face contact. It consisted of five scheduled, structured telephone calls on days three and 10 and then at one, three, and six months after hospital discharge. The phone conversations were based on the findings of a clinical audit of the postoperative needs of patients with colorectal cancer.	Functional Assessment of Cancer Therapy-Colorectal (FACT-C)	There was no significant difference in QOL between the groups at any follow-up time point: one month; three months; or six months.
(Hawkes, Pakenham, Chambers, Patrao, & Courneya, 2014) in Australia[26]	∙Adult patients with a histologically confirmed diagnosis of primary colorectal cancer within the previous 12 months (*n* = 410: intervention *n* = 205 vs. control n = 205) ∙Mean age = 64.9 years for the intervention group and 67.8 years for the control group ∙Women (48.3% for the intervention group and 43.9% for the control group)	This intervention featured four components: 1) 11 telephone-delivered health coaching sessions over six months; 2) a participant handbook; 3) regular motivational postcards; and 4) a pedometer. Both groups also received a quarterly study newsletter	Functional Assessment of Cancer Therapy-Colorectal (FACT-C, version 4) was used to measure QOL at baseline, six months and 12 months.	∙In the intervention group, there significant improvements in quality of life were observed at six and 12 months; physical well-being at six and 12 months; social well-being at six months; emotional well-being at six and 12 months; functional well-being at six and 12 months; and additional well-being at six and 12 months. ∙The intervention group showed significantly higher improvements in physical well-being at six and 12 months than the control group. **However, total quality of life score and subscales were also significantly improved in the control group**

Various types of psychosocial interventions were used, including coaching, telephone interviews, face-to-face counseling, and meetings. The instruments used to measure quality of life also varied, but half of the studies utilized questionnaires developed by the European Organization for Research and Treatment for Cancer. The interventions were delivered to the patients repeatedly from baseline to six months; to examine the long-term effect of the intervention, some studies measured the outcome after the intervention was complete. Finally, for most studies the effect of psychosocial interventions on quality of life was not found to be statistically significant; nevertheless, slight improvements in quality of life were detected in all intervention groups.

**Fig. 2:**
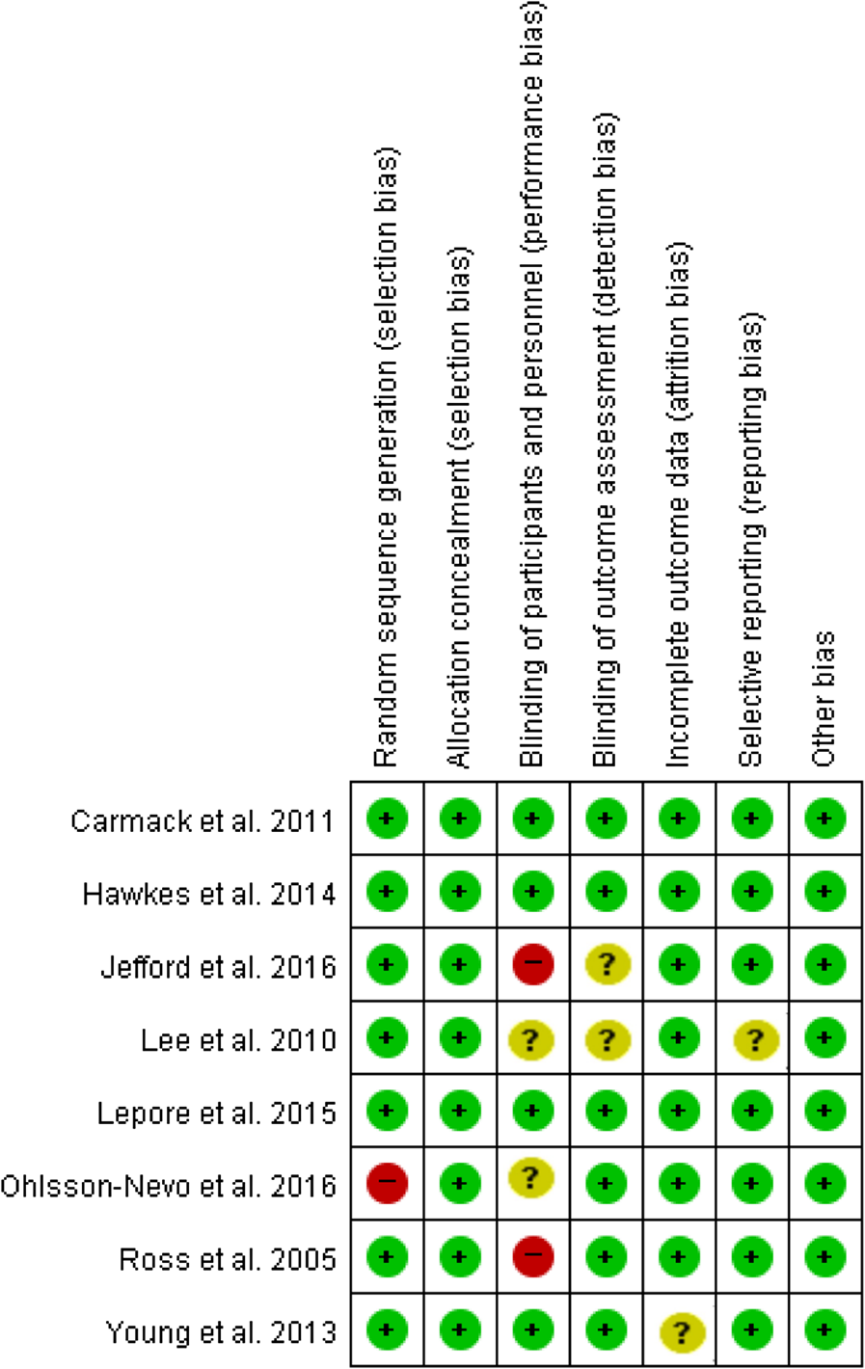
Risk of bias for each included study

We evaluated the overall risk of bias as low. Regarding the individual studies, Fig. 2 shows that the majority of the studies possessed a low risk of bias. Specifically, three studies were evaluated as low risk in all criteria, while the scores for the other studies ranged from four to six.

### Meta-analysis of psychosocial interventions

#### Overall effects

**Fig. 3:**
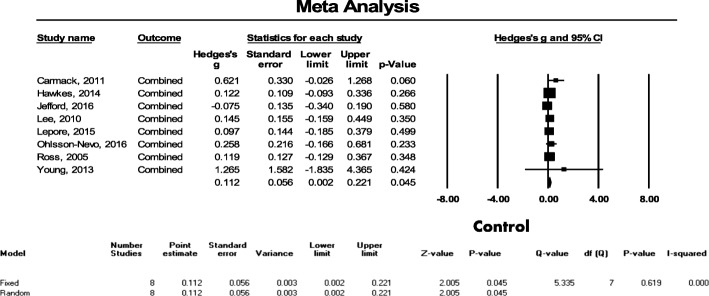
Effect size of psychosocial interventions on overall quality of life

Eight studies featuring a total of 2117 patients were evaluated to examine the effect of psychosocial interventions on the quality of life of CRC patients. In this evaluation, the overall effect of the post-intervention measurement was found to indicate that interventions with no heterogeneity (I^2^ = 0.0%) and a small effect (Hedges’ g = 0.145, SE = 0.056, 95% CI = 0.035–0.254, *p* = 0.009) have a statistically significant benefit on quality of life (see Fig. 3).

Further, the funnel plots were found to be asymmetric for all eight outcomes and the Egger’s test was found to be statistically significant (*p* = 0.012); however, after three studies were filled using the trim and fill method, the adjusted point estimate was 0.143, which indicates that this had no significant effect on the results (Additional file 2). Next, we performed a sensitivity analysis and found that the overall effect size (from 0.131 to 0.155) did not significantly change when any one of the eight studies selected was removed, which means that each individual study had little impact on the overall outcome (Additional file 3).

#### Subgroup analysis

**Fig. 4:**
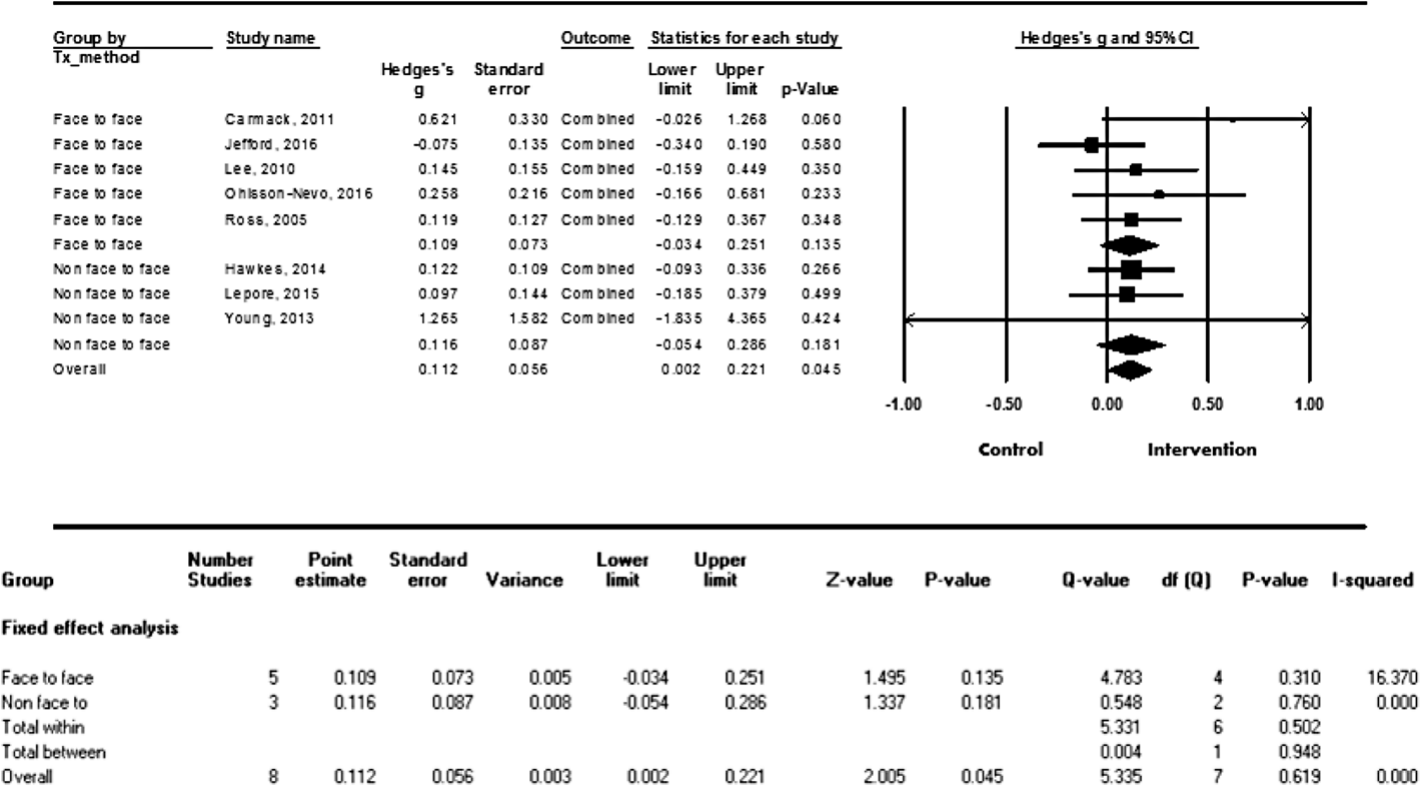
Effect size by types of intervention (face-to-face vs. non-face-to-face)

Since the types and durations of each psychosocial intervention varied, we conducted subgroup analyses to provide more practical evidence. To examine the effectiveness of the specific types of interventions, we categorized them into face-to-face versus non-face-to-face interventions. Five studies used the face-to-face type, and showed a small effect size (g = 0.160, SE = 0.073, 95% CI = 0.018–0.303, *p* = 0.028). The remaining three studies used the non-face-to-face type, and were not significantly associated with improvements in quality of life (g = 0.116, SE = 0.087, 95% CI = − 0.048–0.293, *p* = 0.158) (see Fig. 4).

**Fig. 5:**
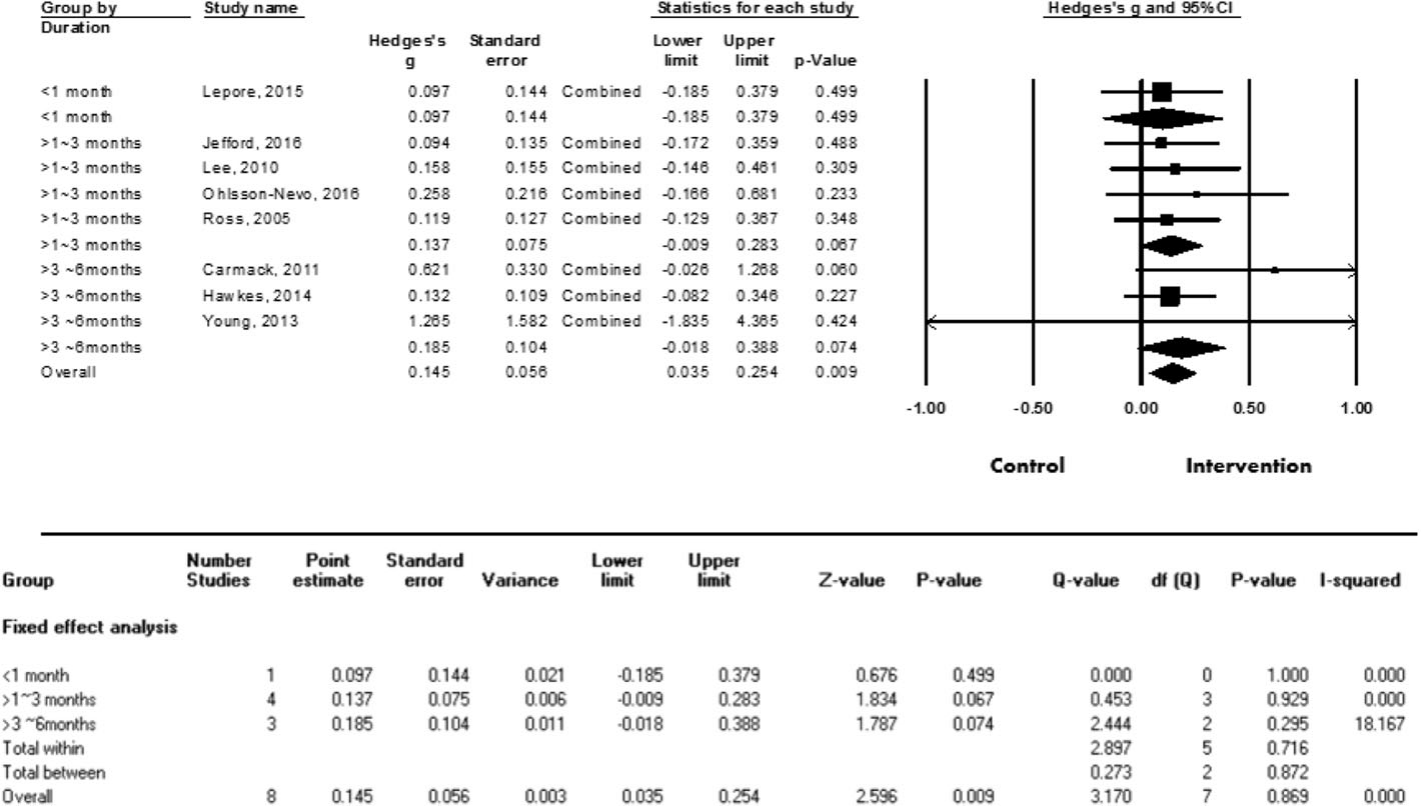
Effect size of meta-analysis on quality of life

To determine the effective intervention duration, the time periods over which the interventions were delivered were categorized into three levels: less than a month; between one and three months; and over three months. As seen in Fig. 5, Hedges’ g was 0.097 for less than one month, 0.137 for between one month and less than three months, and 0.185 for over three months. These findings indicate that the longer the duration of the intervention, the larger the effect size; however, the associations were still not statistically significant (see Fig. 5).

## Discussion

To our knowledge, the current study is the first meta-analysis to provide comprehensive evidence for the effectiveness of psychosocial interventions on the quality of life of CRC patients. A total of eight randomized controlled trials (2117 patients overall) evaluating the application of psychosocial interventions for CRC patients were systematically reviewed, but only one of these studies, that of Hawkes et al. [26], identified a significant improvement in the quality of life of its intervention group.

Our study is comparable with a recent systematic review of 14 randomized controlled trials [17], which showed that three trials found an intervention effect for multiple mental health outcomes, while eight studies exhibited no effect. One clear similarity between our study and that of Mosher et al. is the inclusion of Carmack et al.’s study [27]. Specifically, Carmack et al.’s study [27] revealed that global symptom distress and depression were reduced as a result of their intervention, but quality of life did not improve. In contrast, one important difference between our study and that of Mosher et al. [17] is that they defined the term “psychosocial intervention” in a more comprehensive manner. They stated that it concerns psychotherapy and educational programs, and the researchers also examined both psychosocial factors and quality of life as outcomes, which—along with the use of a greater number of keywords—resulted in a greater number of studies being included.

Although most of the interventions evaluated in the included studies did not have a significant effect on quality of life, the meta-analysis showed that the overall effect at the post-intervention measurement was statistically significant and had a small effect size. This finding is consistent with that of another meta-analysis, which stated that various types of psycho-oncological interventions have significant, small-to-medium beneficial effects on emotional distress and quality of life in adult cancer patients [28]. In contrast, in other meta-analysis studies, such as one that investigated the effectiveness of exercise on the quality of life of CRC patients and one that examined the effect of behavior techniques on the quality of life of breast cancer patients, the respective interventions had no significant short-term effect on quality of life [29, 30]. Although the interventions used in the abovementioned studies might not be comparable (psychosocial vs. exercise), the findings still suggest that psychosocial interventions make a greater contribution to improving quality of life. Considering this, in future studies, various types of interventions should be conducted in order to investigate their effectiveness on quality of life.

### Future clinical implication and research directions

The patients with colorectal cancer more frequently experience psychological distress than do those with other types of cancer [31] and experience social difficulties [32]. Based on our findings, we certainly have strong clinical implications in planning more effective psychosocial interventions. In the current meta-analysis, face-to-face intervention methods showed a statistically significant effect on quality of life, whereas the non-face-to-face intervention, such as email- or telephone-based approaches, did not. Face-to-face interventions appear to enhance the development of therapeutic relationships, thereby leading to an increase in patients’ degree of adherence to healthcare providers’ recommendations. This finding was supported by those of previous studies: compared to Internet-based interventions, face-to-face interventions showed a larger effect size for reducing depressive symptoms [33, 34]. Thus, it is worth using face-to-face methods in clinical settings and they should be considered as the key component of psychosocial interventions for the colorectal cancer survivors.

However, the disadvantages of face-to-face interventions are that they can be time consuming and costly for the patient. As a result, non-face-to-face interventions have been widely used over the past decade. In recent years, Internet/web-based studies have been employed to provide more tailored psychosocial interventions for patients with cancer [35]. Such web-based psychoeducational interventions have been shown to increase the quality of life of family caregivers as well as cancer patients [36], and the development of computer-tailored physical activity interventions for prostate cancer or CRC patients and survivors has now been proposed [37].

In the current research, it was found that the main purpose of psychosocial interventions for colorectal cancer survivors was to improve confidence for self-care through coaching and empowerment [38, 39]. Currently, various types of face-to-face coaching using smart technology have been developed including telephone, video conference, instant message, and email [40, 41]. In addition, the use of virtual reality and augmented reality might be a good alternative to face-to-face interventions because they give the user interactive and instant feedback. For example, virtual reality programs were effective for improving the health-related quality of life of older women [42]. Therefore, it might be beneficial for CRC patients to receive Internet-based, web-based, or virtual reality psychosocial interventions in the future.

The application of smart technology can be a convenient and cost effective method, but the effectiveness should not be compromised for efficiency [43]. As in traditional face-to-face coaching, it is also important to consider the patient’s motivation and level of acceptance of the relationship and establish a relationship based on trust, which is development-oriented, when using smart technology [41, 44].

However, it may not be always possible to utilize those technological resources. In addition, healthcare providers or patients may worry about the technology-based interaction and lose the opportunity to form a relationship or reduce the quality of the relationship. Thus, it is recommended that the healthcare providers use the traditional face-to-face coaching method in an initial psychosocial intervention to develop rapport and interaction with the patients [41]. de Zwaan et al. [45] indicated that the face-to-face therapy was more effective for early treatment than Internet-based guided self-help. Therefore, our finding may guide healthcare professionals in the field by suggesting the use of the face-to-face method at the initial phase of the psychosocial intervention.

We found that the longer the duration of the intervention, the greater the effect size (although this was not statistically significant). This finding is consistent with those of other meta-analysis studies on cancer patients. For example, previous studies have found that longer interventions produce more sustained effects [20, 28], while others found that longer periods of exercise-intervention time are effective for improving quality of life [46]. However, this result should be interpreted cautiously, since only the intervention duration was included in the analysis and the magnitude of the interventions in question (i.e., the frequency and amount of intervention) was not considered. Therefore, further study is required to examine the effect size in terms of intervention duration and strength. Furthermore, it is necessary to conduct larger randomized controlled trials for longer durations in the future in order to identify the most effective time period for the improvement of quality of life.

To increase the level of evidence for the current meta-analysis, only randomized controlled trial studies were included and the quality assessment was undertaken using the Cochrane Collaboration’s risk of bias tool. First, at the random-sequence-generation domain, as the randomization methods were clearly described in the text, a total of seven studies were evaluated as having a low risk of bias. As for incomplete outcome data, most studies were graded as having a low risk of bias with low dropout rates. Next, regarding the blinding of participants and personnel, two studies were evaluated as having a high risk of bias. Finally, for blinding the outcome assessment, six studies were rated as having a low risk of bias. It should be noted that the use of inadequate blinding may produce detection bias, which means that the measured values might not reflect reality. Considering the above, future research should underline allocation concealment, blinding of participants and personnel, and outcome assessors in order to obtain more reliable conclusions.

### Study limitations

There are several limitations in the current meta-analysis. First, although we used thorough search strategies to minimize inclusion and publication bias, there is still the possibility that some studies were missed. Second, the number of studies included in the current meta-analysis is relatively small, and the characteristics of the patients in each study were also heterogeneous; therefore, it may be premature to generalize these findings. It is necessary to focus on delineating the key characteristics of the psychosocial interventions found to be effective by using a prospective design. Third, the current study applied the total score for quality of life in the meta-analysis, since the instruments used to measure quality of life varied for each study. Psychosocial interventions might be more effective for certain subdomains of quality of life. Thus, future studies are required to investigate the effect of the interventions on specific aspects of the quality of life construct. Although the majority of studies measured the outcomes multiple times through follow-up assessments, longitudinal studies over the course of randomized controlled trials were found to be insufficient. As such, future studies should employ a larger randomized controlled trial using a longitudinal study design.

## Conclusions

This meta-analysis provides evidence for the beneficial effect of face-to-face-based psychosocial interventions on the quality of life of CRC patients. Of course, it is still premature to generalize these findings, as the identified effect size was small. Given that quality of life changes with time and emphasizes the importance of personal growth [47], it may take time to fully detect improvements in quality of life. Further, the quality of life of cancer patients may also depend on the prognosis during the disease trajectory. To improve quality of life for CRC patients, therefore, further studies should assess physical functioning or the severity of symptoms before face-to-face intervention methods are utilized.

## Additional files

Additional file 1: Search strategies for used database. (DOCX 21 kb)

Additional file 2: Funnel plot of Hedge’s g against its standard error for quality of life using trim and fill method. (DOCX 126 kb)

Additional file 3: Overall effect size when one study was removed. (DOCX 25 kb)

## Abbreviations

CI: Confidence interval
CRC: Colorectal cancer
