# Supplementary material for: Effect of psychosocial interventions on the quality of life of patients with colorectal cancer: a systematic review and meta-analysis
Source: Health Qual Life Outcomes. 2018 Jun 8;16:119. doi: 10.1186/s12955-018-0943-6 (PMC5994008; doi:10.1186/s12955-018-0943-6)
Supplement: Supplementary file 1 — Search strategies for used database. (DOCX 21 kb) [file 12955_2018_943_MOESM1_ESM.docx]

**Additional file 1. Search strategies for used database**

| **PubMed** | #1 | "Colorectal Neoplasms"[Mesh] OR "Colorectal cancer"[All Fields] OR "Colorectal cancers"[All Fields] OR "colorectal neoplasm"[All Fields] OR "colorectal neoplasms"[All Fields] OR "colorectal tumour"[All Fields] OR "colorectal tumours"[All Fields] OR "colorectal tumor"[All Fields] OR "colorectal tumors"[All Fields] |
| --- | --- | --- |
|  | #2 | "Randomized Controlled Trial"[Publication Type] OR "Randomized Controlled Trials as Topic"[Mesh] OR "Randomized Controlled Trial"[All Fields] OR "Comparative Study"[Publication Type] OR "Comparative Study"[All Fields] OR "Non-Randomized Controlled Trials as Topic"[Mesh] OR "Non-Randomized Controlled Trials"[All Fields] OR "quasi experimental study"[All Fields] OR "pre post study"[All Fields] |
|  | #3 | "Quality of Life"[Mesh] OR "Quality of Life"[All Fields] OR "well-being"[All Fields] OR "psychological outcome"[All Fields] |
|  | #4 | "Education"[Mesh] OR Education OR "Counseling"[Mesh] OR Counseling OR "Nursing"[Mesh] OR Nursing OR intervention OR psychoeducation OR psychoeducational OR "Behavior Therapy"[Mesh] OR "Behavior Therapy" OR "Behavioral Therapy" OR "Behavioural Therapy" OR "Cognitive Therapy"[Mesh] OR "cognitive behavioural therapy" OR "cognitive behavioral therapy" OR "communication skills training" OR "Self-Help Groups"[Mesh] OR "Self-Help Groups" OR "support group" OR "problem solving therapy" OR "Motor Activity"[Mesh] OR "Motor Activity" OR "physical activity" OR nutrition OR nutritional OR "expressive writing" |
|  | **#5** | **#1 AND #2 AND #3 AND #4** |
|  |  |  |
| **EMBASE** | #1 | colorectal neoplasia' OR 'colorectal neoplasm' OR 'colorectal neoplasms' OR 'colorectal tumor' OR 'colorectal tumour' OR 'tumor, colorectal' OR 'tumour, colorectal' OR 'colorectal cancer' OR 'colorectal cancers' OR 'colorectal tumors' OR 'colorectal tumours' |
|  | #2 | randomized controlled trial'/exp OR 'controlled trial, randomized' OR 'pragmatic clinical trial' OR 'pragmatic clinical trials' OR 'randomised controlled study' OR 'randomised controlled trial' OR 'randomized controlled study' OR 'randomized controlled trial' OR 'trial, randomized controlled' OR 'randomized controlled trial (topic)'/exp OR 'pragmatic clinical trials as topic' OR 'randomized controlled trial (topic)' OR 'randomized controlled trials' OR 'randomized controlled trials as topic' OR 'comparative study'/exp OR 'comparative studies' OR 'comparative study' OR 'comparison' OR 'quasi experimental study'/exp OR 'quasi experimental study' OR 'quasiexperimental study' OR 'pre-post study' |
|  | #3 | quality of life'/exp OR 'hrql' OR 'health related quality of life' OR 'life quality' OR 'quality of life' OR 'wellbeing'/exp OR 'well being' OR 'wellbeing' OR 'wellness' OR 'psychological well being'/exp OR 'psychological well being' OR 'psychological wellbeing' OR 'psychological outcome' OR 'psychological outcomes' |
|  | #4 | education'/exp OR 'baccalaureate education' OR 'child education' OR 'college admission test' OR 'education' OR 'education of mentally retarded' OR 'education service' OR 'education, distance' OR 'education, nonprofessional' OR 'education, pharmacy' OR 'education, pharmacy, continuing' OR 'education, pharmacy, graduate' OR 'education, special' OR 'education, veterinary' OR 'educational measurement' OR 'internship, nonmedical' OR 'perceptorship' OR 'pharmacy residencies' OR 'preceptorship' OR 'school admission criteria' OR 'self-evaluation programmes' OR 'self-evaluation programs' OR 'training support' OR 'counseling'/exp OR 'client centered therapy' OR 'counseling' OR 'counselling' OR 'nondirective therapy' OR 'pastoral care' OR 'person centered therapy' OR 'person-centered therapy' OR 'nursing'/exp OR 'nursing' OR 'nursing audit' OR 'nursing service' OR 'nursing service, hospital' OR 'nursing services' OR 'nursing support' OR 'nursing, private duty' OR 'nursing, supervisory' OR 'nursing, team' OR 'office nursing' OR 'private duty nursing' OR 'intervention' OR 'psychoeducation'/exp OR 'psychoeducation' OR psychoeducational OR 'behavior therapy'/exp OR 'behavior therapy' OR 'behavior training' OR 'behavior treatment' OR 'behavioral therapy' OR 'behaviour therapy' OR 'behaviour training' OR 'behaviour treatment' OR 'behavioural therapy' OR 'desensitisation (psychology)' OR 'desensitisation, psychologic' OR 'desensitization (psychology)' OR 'desensitization, psychologic' OR 'eye movement desensitisation and reprocessing' OR 'eye movement desensitisation reprocessing' OR 'eye movement desensitization and reprocessing' OR 'eye movement desensitization reprocessing' OR 'implosive therapy' OR 'therapy, behavior' OR 'therapy, behaviour' OR 'treatment, behavior' OR 'treatment, behaviour' OR 'cognitive therapy'/exp OR 'cbt (cognitive behavioral therapy)' OR 'cbt (cognitive behavioural therapy)' OR 'cognitive behavior therapy' OR 'cognitive behavior treatment' OR 'cognitive behavioral therapy' OR 'cognitive behavioral treatment' OR 'cognitive behaviour therapy' OR 'cognitive behaviour treatment' OR 'cognitive behavioural therapy' OR 'cognitive behavioural treatment' OR 'cognitive therapy' OR 'communication skills training' OR 'support group'/exp OR 'group, support' OR 'support group' OR 'problem solving therapy' OR 'physical activity'/exp OR 'activity, physical' OR 'physical activity' OR 'nutrition'/exp OR 'diet, food, and nutrition' OR 'nutrition' OR 'nutrition council' OR 'nutrition phenomena' OR 'nutrition physiology' OR 'nutrition processes' OR 'nutrition research' OR 'nutrition research center' OR 'nutrition study' OR 'nutrition survey' OR 'nutrition surveys' OR 'nutritional physiological phenomena' OR 'nutritional physiology' OR 'nutritive solution' OR 'sports nutritional physiological phenomena' OR nutritional OR 'expressive writing' |
|  | **#5** | **#1 AND #2 AND #3 AND #4** |
|  |  |  |
| **CINAHL** | #1 | (MH "Colorectal Neoplasms+") OR "colorectal tumours" OR "colorectal tumour" OR "colorectal neoplasm" OR "colorectal neoplasms" OR "colorectal cancer" OR "colorectal cancers" OR "colorectal tumor" OR "colorectal tumors" |
|  | #2 | (MH "Randomized Controlled Trials") OR "Randomized controlled trial" OR (MH "Comparative Studies") OR "comparative study" OR "Comparative Studies" OR (MH "Quasi-Experimental Studies+") OR "quasi-experimental study" OR "pre-post study" |
|  | #3 | (MH "Quality of Life+") OR "Quality of life" OR "well-being" OR (MH "Psychological Well-Being") OR "psychological well-being" OR "psychological outcome" OR "psychological outcomes" |
|  | #4 | (MH "Education+") OR education OR (MH "Counseling+") OR counseling OR "nursing" OR "intervention" OR (MH "Psychoeducation") OR "Psychoeducation" OR "Psychoeducational" OR (MH "Behavior Therapy+") OR "Behavior Therapy" OR "behavioral therapy" OR behavioural therapy" OR (MH "Cognitive Therapy+") OR "Cognitive Therapy" OR "cognitive behavioral therapy" OR "cognitive behavioural therapy" OR (MH "Communication Skills Training") OR "Communication Skills Training" OR (MH "Support Groups+") OR "Support Groups" OR "support group" OR "problem solving therapy" OR (MH "Physical Activity") OR "physical activity" OR (MH "nutrition+") OR nutrition OR nutritional OR "expressive writing" |
|  | **#5** | **#1 AND #2 AND #3 AND #4** |
|  |  |  |
| **Cochrane** | #1 | MeSH descriptor: [Colorectal Neoplasms] explode all trees OR "colorectal tumours" or "colorectal tumour" or "colorectal neoplasm" or "colorectal neoplasms" or "colorectal cancer" or "colorectal cancers" or "colorectal tumor" or "colorectal tumors" |
|  | #2 | MeSH descriptor: [Randomized Controlled Trial] explode all trees OR MeSH descriptor: [Randomized Controlled Trials as Topic] explode all trees OR "Randomized controlled trial" OR  MeSH descriptor: [Comparative Study] explode all trees OR "comparative study" OR "Comparative Studies" OR MeSH descriptor: [Non-Randomized Controlled Trials as Topic] explode all trees OR "quasi-experimental study" OR "pre-post study" |
|  | #3 | MeSH descriptor: [Quality of Life] explode all trees OR "Quality of life" OR "well-being" OR "psychological well-being" OR "psychological outcome" OR "psychological outcomes" |
|  | #4 | MeSH descriptor: [Education] explode all trees OR Education OR MeSH descriptor: [Counseling] explode all trees OR counseling OR nursing OR intervention OR psychoeducation OR psychoeducational OR "behavioural therapy" OR "behavioural therapy" OR "cognitive behavioural therapy" OR "cognitive behavioral therapy" OR "communication skills training" OR MeSH descriptor: [Self-Help Groups] explode all trees OR "support group" OR "problem solving therapy" OR MeSH descriptor: [Motor Activity] explode all trees OR "Motor Activity" OR "physical activity" OR nutrition OR nutritional OR "expressive writing" |
|  | **#5** | **#1 AND #2 AND #3 AND #4** |
|  |  |  |
| **PsycArticles** | #1 | "colorectal neoplasia" OR "colorectal neoplasm" OR "colorectal neoplasms" OR "colorectal tumor" OR "colorectal tumour" OR "tumor, colorectal" OR "tumour, colorectal" OR "colorectal cancer" OR "colorectal cancers" OR "colorectal tumors" OR "colorectal tumours" |
|  | #2 | "randomized controlled trial" OR "controlled trial, randomized" OR "pragmatic clinical trial" OR "pragmatic clinical trials" OR "randomised controlled study" OR "randomised controlled trial" OR "randomized controlled study" OR "trial, randomized controlled" OR "pragmatic clinical trials" OR "randomized controlled trials" OR "comparative study" OR "comparative studies" OR "quasi experimental study" OR "quasiexperimental study" OR "pre-post study" |
|  | #3 | "quality of life" OR "health related quality of life" OR "life quality" OR "wellbeing" OR "well being" OR "wellness" OR "psychological well being" OR "psychological wellbeing" OR "psychological outcome" OR "psychological outcomes" |
|  | #4 | Education OR counseling OR nursing OR intervention OR psychoeducation OR psychoeducational OR “behavioural therapy” OR “behavioral therapy” OR “cognitive behavioral therapy” OR “cognitive behavioural therapy” OR “communication skills training” OR “support group” OR “problem solving therapy” OR “physical activity” OR “Motor Activity” OR nutrition OR nutritional OR “expressive writing” |
|  | **#5** | **#1 AND #2 AND #3 AND #4** |
|  |  |  |
| **Web of Science** | #1 | "colorectal neoplasia" OR "colorectal neoplasm" OR "colorectal neoplasms" OR "colorectal tumor" OR "colorectal tumour" OR "tumor, colorectal" OR "tumour, colorectal" OR "colorectal cancer" OR "colorectal cancers" OR "colorectal tumors" OR "colorectal tumours" |
|  | #2 | "randomized controlled trial" OR "controlled trial, randomized" OR "pragmatic clinical trial" OR "pragmatic clinical trials" OR "randomised controlled study" OR "randomised controlled trial" OR "randomized controlled study" OR "trial, randomized controlled" OR "pragmatic clinical trials" OR "randomized controlled trials" OR "comparative study" OR "comparative studies" OR "quasi experimental study" OR "quasiexperimental study" OR "pre-post study" |
|  | #3 | "quality of life" OR "health related quality of life" OR "life quality" OR "wellbeing" OR "well being" OR "wellness" OR "psychological well being" OR "psychological wellbeing" OR "psychological outcome" OR "psychological outcomes" |
|  | #4 | Education OR counseling OR nursing OR intervention OR psychoeducation OR psychoeducational OR “behavioural therapy” OR “behavioral therapy” OR “cognitive behavioral therapy” OR “cognitive behavioural therapy” OR “communication skills training” OR “support group” OR “problem solving therapy” OR “physical activity” OR “Motor Activity” OR nutrition OR nutritional OR “expressive writing” |
|  | **#5** | **#1 AND #2 AND #3 AND #4** |
|  |  |  |
| **SCOPUS** | #1 | "colorectal neoplasia" OR "colorectal neoplasm" OR "colorectal neoplasms" OR "colorectal tumor" OR "colorectal tumour" OR "tumor, colorectal" OR "tumour, colorectal" OR "colorectal cancer" OR "colorectal cancers" OR "colorectal tumors" OR "colorectal tumours" |
|  | #2 | "randomized controlled trial" OR "controlled trial, randomized" OR "pragmatic clinical trial" OR "pragmatic clinical trials" OR "randomised controlled study" OR "randomised controlled trial" OR "randomized controlled study" OR "trial, randomized controlled" OR "pragmatic clinical trials" OR "randomized controlled trials" OR "comparative study" OR "comparative studies" OR "quasi experimental study" OR "quasiexperimental study" OR "pre-post study" |
|  | #3 | "quality of life" OR "health related quality of life" OR "life quality" OR "wellbeing" OR "well being" OR "wellness" OR "psychological well being" OR "psychological wellbeing" OR "psychological outcome" OR "psychological outcomes" |
|  | #4 | Education OR counseling OR nursing OR intervention OR psychoeducation OR psychoeducational OR “behavioural therapy” OR “behavioral therapy” OR “cognitive behavioral therapy” OR “cognitive behavioural therapy” OR “communication skills training” OR “support group” OR “problem solving therapy” OR “physical activity” OR “Motor Activity” OR nutrition OR nutritional OR “expressive writing” |
|  | **#5** | **#1 AND #2 AND #3 AND #4** |
