# Supplementary material for: Effect of psychosocial interventions on the quality of life of patients with colorectal cancer: a systematic review and meta-analysis
Source: Health Qual Life Outcomes. 2018 Jun 8;16:119. doi: 10.1186/s12955-018-0943-6 (PMC5994008; doi:10.1186/s12955-018-0943-6)

Additional file 2. Funnel plot of Hedge’s g against its standard error for quality of life using trim and fill method


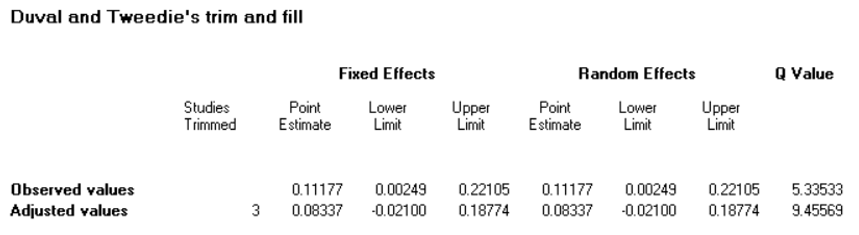

Supplement: Supplementary file 2 — Funnel plot of Hedge’s g against its standard error for quality of life using trim and fill method. (DOCX 126 kb) [file 12955_2018_943_MOESM2_ESM.docx]
