# Supplementary material for: Effect of psychosocial interventions on the quality of life of patients with colorectal cancer: a systematic review and meta-analysis
Source: Health Qual Life Outcomes. 2018 Jun 8;16:119. doi: 10.1186/s12955-018-0943-6 (PMC5994008; doi:10.1186/s12955-018-0943-6)
Supplement: Supplementary file 3 — Overall effect size when one study was removed. (DOCX 25 kb) [file 12955_2018_943_MOESM3_ESM.docx]

Additional file 3. Overall effect size when one study was removed
